# The use of disaggregate data in evaluations of public health interventions: cross-sectional dependence can bias inference

**DOI:** 10.1186/s13690-022-00795-5

**Published:** 2022-01-20

**Authors:** Torleif Halkjelsvik, Antonio Gasparrini, Rannveig Kaldager Hart

**Affiliations:** 1 Centre for Evaluation of Public Health Measures, Norwegian Institute of Public Health, Folkehelseinstituttet, Postboks 222 Skøyen, 0213 Oslo, Norway; 2 Department of Alcohol Tobacco and Drugs, Norwegian Institute of Public Health, Oslo, Norway; 3 Department of Public Health Environments and Society, London School of Hygiene & Tropical Medicine, London, UK; 4 Centre for Statistical Methodology, London School of Hygiene & Tropical Medicine, London, UK; 5 Department of Social Inequality, Norwegian Institute of Public Health, Oslo, Norway

**Keywords:** Cross-sectional dependence, Contemporaneous error, Public health, Intervention, Interrupted time series, Segmented regression, Disaggregate data, Individual-level data

## Abstract

Higher availability of administrative data and better infrastructure for electronic surveys allow for large sample sizes in evaluations of national and other large scale policies. Although larger datasets have many advantages, the use of big disaggregate data (e.g., on individuals, households, stores, municipalities) can be challenging in terms of statistical inference. Measurements made at the same point in time may be jointly influenced by contemporaneous factors and produce more variation across time than suggested by the model. This excess variation, or co-movement over time, produce observations that are not truly independent (i.e., cross-sectional dependence). If this dependency is not accounted for, statistical uncertainty will be underestimated, and studies may indicate reform effects where there is none. In the context of interrupted time series (segmented regression), we illustrate the potential for bias in inference when using large disaggregate data, and we describe two simple solutions that are available in standard statistical software.

Electronic surveys, technology such as bar code scanners, and administrative (register) data allow for huge sample sizes in evaluations of public health policies. For example, a study on the impact of excise tax on sugar-sweetened beverages included data from over 15 million checkouts from stores in Berkeley [1]. Larger datasets can increase precision and provide higher power to detect effects of public health measures. However, as we will demonstrate below, more data is not always better, and sometimes it can produce a false impression of high precision.

In the evaluation of public health interventions, one often has the option to collapse data across units and analyse changes at the aggregate level, such as proportion of smokers or the total sales of unhealthy products. The problem described in the present article does not apply to these types of analyses; it applies to evaluations using unit-level data (e.g., on individuals, households or municipalities). Such disaggregate data are often used when unit-level covariates are included in the analysis, for example, to control for compositional changes or to test for moderation by variables such as socioeconomic status or geography. In these cases, the increased precision from large samples may be illusive because there are dependencies between units measured at the same point in time. This is particularly a problem when a policy is introduced for a large proportion of—or typically the entire—sample at the same point in time (e.g., the introduction of excise taxes, restrictions on the availability of a product, health warning labels).

Below we will first describe how the problem of dependence between units measured at the same point in time can be considered as a problem of excess variation (similar to the notion of overdispersion), then we provide indications that this problem is neglected in analyses on disaggregate data in the literature on public health interventions. We provide an empirical example that demonstrate how severe the problem can be, along with two simple solutions that utilize methods that are familiar to the applied researcher. We will focus on the use of disaggregate data in the design typically referred to as interrupted time series (ITS) or segmented regression, but our point is highly relevant also in designs involving non-equivalent control groups.

## Unmodelled common influences produce excess variation

There are many problems in causal inference when the data is observational and when a policy is introduced at one specific point in time [2]. One problem that is of particular relevance when datasets grow bigger is unmodelled common influences on data sampled at the same point in time. Such influences could be the weather, major social events, news, marketing, research methods (interviewer effect, selective sampling), contagious diseases, changes in the economy, social network effects, or fads and fashions. In disaggregate data (e.g., repeated cross-sectional and panel data on individuals), the excess variation from unmodelled common influences produce a form of dependency between units that is often referred to as cross-sectional dependency or contemporaneous correlation. When observations are not truly independent, standard measures of statistical uncertainty will typically be biased downwards (i.e., the uncertainty of the result is underestimated).

In analyses on aggregate data (e.g., aggregate time series of total consumption in a country) there cannot be a problem of dependence between observations within each time point because there is only one observation per time point. However, unmodelled common influences on the underlying sample from which the aggregate measure is derived can still produce excess variation if the variance is pre-determined by the statistical model, such as in standard count and binomial models. In these models, excess variation is referred to as overdispersion, which may be a more familiar concept than cross-sectional dependence. Overdispersion is typically not considered as relevant in disaggregate analyses, for example in analyses on binary responses [3]. Yet, if an analysis on aggregated data in a logistic binomial regression indicates overdispersion, one cannot avoid the problem by running a logistic regression on individual-level data [4].

Both overdispersion and the type of cross-sectional dependence discussed in the present article reflect influences on data that increase variance beyond mere sampling error (note that there also exists other forms of cross-sectional dependence and overdispersion, e.g., sub-cluster dependencies, and overdispersion not related to time of measurement). This excess variation can be considered as a misspecification problem, for example, due to missing variables in the model. As such, it should motivate more rigorous modelling of factors that can influence the outcome of interest. However, it is often impossible to fully model a process that unfolds over time, and it has been argued that excess variation is the rule rather than the exception [5 (p.124)]. Thus, unless one can avoid conducting the analysis altogether, one needs to assume that, after careful modelling, the remaining excess variation is random.

In count and proportion models on aggregate time series, there are a range of ways to account for excess random variation, such as scaling the standard errors of the coefficients by an overdispersion parameter or by directly modelling the overdispersion (e.g., beta-binomial models, observation-level-random effects; see [6]). In the case of disaggregate data, several solutions to the related problem of cross-sectional dependence have been proposed, particularly in the context of panel models in the econometric literature [7–11]. Below we will provide an empirical example of cross-sectional dependence and suggest how to accommodate the problem. Before the empirical example, we briefly present indications that this issue may have been neglected in applied research on public health interventions.

## Is there a problem?

We inspected the primary studies of a recent meta-analysis of studies on sugary beverage tax [12]. Two of the studies did not include inferential statistics, three investigated multiple within-state changes, and one was a cross-sectional comparison, but of the thirteen remaining studies, ten studies did not adjust or test for excess variation over time. One study accounted for heterogeneity in the time dimension in the selection of control group, one study used aggregate data, and only one study adjusted standard errors for clustering on the time dimension.

Based on the original data of a recent review of interrupted time series studies in epidemiology [13], we noted the following pattern: Of fourteen ITS studies where the data was based on binary outcomes and the analysis was a logistic regression (including GEE and GLMM with logit link), twelve studies used individual-level data and did not adjust or test for excess variation (i.e., cross-sectional dependence/residual heterogeneity). One of two studies that used aggregate data adjusted for overdispersion. Of thirteen studies where the underlying data was similarly based on binary outcomes but the analysis was variations of Poisson regressions (on aggregate data except for one individual-level study), seven studies accounted for overdispersion by random intercept of time, quasi-poisson, or the use of robust standard errors. The above suggests that overdispersion is often a concern in aggregate analyses, whereas cross-sectional dependence is not a concern in individual-level analyses. Below we provide an example of why the latter could be a problem.

## An illustrative case: The potential effect of a sugary product tax

When analyzing aggregate data with a linear regression model, it is largely inconsequential whether the variation in the data is due to the random variation between and within the underlying observations (e.g., individuals) or due to random common influences (such as the weather, sports events, marketing campaigns, variations in research methods, etc.). The error term in a linear interrupted time series on aggregate data reflects any source of variation, and inference is based on this overall variation. For analyses on disaggregate data, when measurements of several units are made at multiple time points, inference is less straightforward.

To illustrate this point, we present analyses using cluster-robust standard errors with time as a grouping factor. Applied researchers habitually apply heteroscedastic robust standard errors (Eiker-Huber-White or “sandwich” estimator), and also its generalization, the Cluster-Robust Standard Errors, where the robust standard error is calculated on residuals collapsed by clusters [8]. However, the clustering variable in public health research is typically the cross-sectional unit such as geographical regions, individuals, households, etc. Below we use a version of cluster-robust standard errors that allows for clustering on both the cross-sectional units (in our case grocery stores) and the time dimension (week). See reference 7 for a detailed description of such multi-way cluster-robust standard errors.

**Fig. 1:**
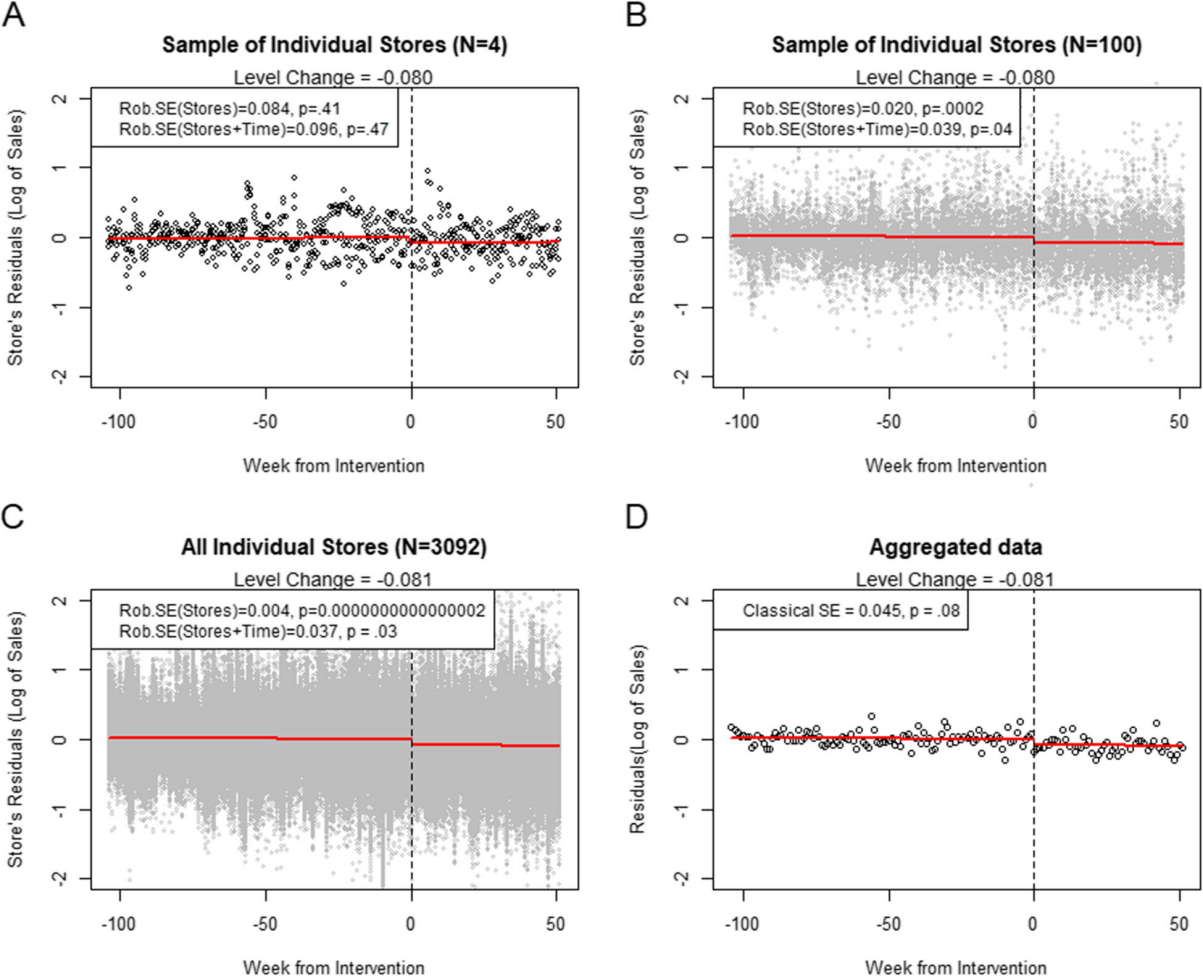
The effect of ignoring the time variable in analyses of policy change on disaggregate data. The Cluster-Robust Standard Error (Rob.SE) with clustering on Stores gives increasingly biased standard errors as a function of increasing sample size (panels **A** to **C**). The Rob. SE with clustering on both Stores and Time gives results in line with the analysis on aggregated data (panel **D**)

Figure 1 present analyses of sales data from two years before to one year after the implementation of a substantial increase in the sugary product tax in Norway. It shows the residuals of the log-transformed sales of chocolate plates after accounting for the following variables at the level of individual grocery stores: intercepts, seasonality (week number), moving holidays, linear time trends and level change from before to after the intervention. For the purpose of illustrating the potential effect of the intervention, the trend and level change terms were added back into the residuals and drawn as lines (i.e., the graphs are not partial regression plots, but plots that maintain the chronology of time).

In Panel A, where a sample of 4 stores have been drawn from the dataset, the standard errors and p-values of a model with cluster-robust standard errors at the level of stores (one-way clustering) are not different from a model with clustering on both store and time (two-way clustering). In Panel B, with 100 stores, the discrepancy between the one-way and two-way clustered models becomes larger, and in Panel C, using data from 3092 stores, the difference between the one-way and two-way clustered standard errors is extremely large. The aggregated data in Panel D is based on the same 3092 stores as Panel C and therefore carries the same amount of information in terms of the mean changes from pre to post. Accordingly, the standard error of the aggregated analysis (a standard interrupted time series model), is fairly similar to the disaggregate analyses with two-way clustered standard errors. As can be observed by the two-way cluster-robust standard errors in Panels B and C, a 30 times increase in sample size from 100 to 3092 stores did not increase precision of our effect estimate when accounting for clustering on the time dimension, but it substantially increased the precision of the analysis clustered only on the cross-sectional units.

As this example illustrates, cluster robust standard errors grouped on both time and cross-sectional unit are inflated relative to the standard errors grouped on the cross-sectional unit only. This inflation compensates for the “fake precision” given by a large sample when there is dependence within measurements. The amount of inflation is a function of the within-cluster correlation of the predictor (which, in terms of time clusters, is as high as 1 for a term representing a policy that is introduced at a specific point in time), the within-cluster correlation of the residuals, and the number of observations within each cluster [8]. After this inflation, standard errors again give a better estimate of statistical uncertainty for the specific model, also when the observations within the clusters are not truly independent.

Note that when the sample size is very large and the number of measurements across time is limited, it may not even be necessary to cluster on the cross-sectional units [14]. In supplemental analyses (not reported), one-way SE clustered on time (not on stores) gave similar results as the two-way robust SE (stores + time) for the data presented in Panel B and C, but not Panel A.

When robust and classical standard errors differ, this can be interpreted as a sign of potential model misspecification and should motivate more rigorous modelling [15]. When the excess variation is not random but systematically related to the impact variable, better modelling is required to obtain unbiased estimates of the impact of a policy. Better modelling is also an issue of statistical power. In the example presented in Fig. 1, 3000 stores do not really provide more information than 100 stores, suggesting that there is little to be gained from increasing the sample even further. Increasing the sample on the time axis can be risky in interrupted time series, because secular trends may change. Sometimes, the statistical power of evaluations can only be increased by better modelling—not by sampling additional time points or cross-sectional units.

## Multilevel modelling and other approaches

If only a few pre- and post measurements are available, the two-way cluster-robust standard errors will be biased downwards. There are numerous attempts to correct for bias in cluster-robust standard errors [8, 16, 17], but in common statistical software, few methods are implemented for two-way clustering (see e.g., [18] for a user-written function in Stata). Another alternative based on a tool commonly used by applied researchers, is to model the grouping factor (i.e., time) as a random intercept in mixed models (multilevel models).

In contrast to the cluster-robust SE correction, which account for any cross-sectional dependence within the measurements at each time points, random intercepts of time only account for the macro-level co-movements, with the additional assumptions that the distribution of the excess variation has a certain shape. In principle, this means that the multilevel models have stronger assumptions than the OLS models with cluster-robust SEs. However, due to the bias in cluster-robust SEs when there are few clusters, the mixed models are often more conservative than the cluster-robust SEs. In the present examples in Fig. 1, using a linear mixed modelling approach, the standard error for analysis of the data in Panel A is 0.060 (versus 0.096 for two-way cluster-robust), for Panel B the standard error is 0.045 (versus 0.039) and for Panel C the standard error is 0.045 (versus 0.037).

Note that there are more specialized and comprehensive ways of analyzing the type of data we have used. For instance, dependence within time clusters can be modelled as regional interdependencies [19], and potential serial correlation can be modelled at the level of residuals or as common persistent influences [14, 20]. We have focused on the use of multilevel modelling and cluster-robust standard errors because these are included in the basic statistical toolkits of many applied researchers. Furthermore, multilevel modelling and cluster-robust standard errors are all-round tools that can address cross-sectional dependence in panel/longitudinal data, repeated cross-sections, and even overdispersion in aggregate count and proportion data (s﻿ee [ 3,﻿ 4]).

## Conclusions

When a large number of units are sampled at the same point in time, cross-sectional dependence can produce bias in standard errors and p-values. As an illustration of this, we provided a simplified case using chocolate sales data before and after an increase in tax. Due to a high level of heterogeneity across time, our example gave large differences in inferential statistics between standard analyses and analyses that accounted for cross-sectional dependence. Similar levels of bias in inferential statistics may be achieved with less heterogeneity but larger samples (e.g., population register data).

Note that this was not a best practice tutorial for the analysis of public health interventions. There are certainly other threats to estimation and inference when using observational data. Our goal was simply to illustrate how excess variation over time can produce severely biased inference and to show how the inclusion of time as a grouping factor can detect and prevent this. The reporting of cluster-robust SEs or random intercepts grouped on the time dimension should not be used as a substitution for rigorous modelling.

## Data Availability

Data are provided by The Nielsen Company, Norway. The authors are not entitled to share the data.

## Abbreviations

SE: Standard Error
ITS: interrupted times series
